# Neonatal supraventricular tachycardia: current diagnostic approaches and emerging technologies

**DOI:** 10.3389/fped.2025.1694215

**Published:** 2026-01-05

**Authors:** Jianhong Qi, Ruihua Yu, Xiaokang Wang

**Affiliations:** Department of Neonatology, Shandong Provincial Hospital Affiliated to Shandong First Medical University, Jinan, Shandong, China

**Keywords:** artificial intelligence, electrocardiography, machine learning, neonatal arrhythmia, supraventricular tachycardia, telemedicine, wearable sensors

## Abstract

Neonatal supraventricular tachycardia (SVT) represents the most common pathological tachyarrhythmia in the neonatal period, with an incidence of 1:250–1000 live births. This review synthesizes current diagnostic methodologies and explores the potential of emerging technological innovations. Traditional modalities, including 12-lead electrocardiography (ECG) and ambulatory monitoring, remain foundational but face limitations regarding signal quality and intermittent capture in neonates. Emerging technologies—specifically deep learning algorithms, biocompatible wearable sensors, and non-contact sensing modalities—offer promising avenues to enhance detection. While AI models in broader pediatric cohorts have reported diagnostic accuracies exceeding 90%, neonatal-specific validation remains a critical need. This review discusses the integration of these tools into clinical workflows, highlighting potential improvements in diagnostic timing while addressing persistent technical, regulatory, and ethical barriers. We provide a framework for clinicians navigating this evolving landscape, emphasizing the need for rigorous validation of new technologies in the unique neonatal population.

## Highlights

- First comprehensive synthesis bridging traditional diagnostic approaches with cutting-edge technologies (AI algorithms, wearable biosensors, non-contact monitoring) for neonatal SVT, providing clinicians with an evidence-based framework for integrating innovations that achieve >90% diagnostic accuracy.
- Real-world implementation data from early-adopter institutions demonstrating 50% reduction in diagnostic delays and practical strategies for overcoming technical, regulatory, and socioeconomic barriers in diverse clinical settings.
- Specific research priorities and implementation roadmap addressing critical gaps in neonatal cardiac monitoring, where current systems show <1% positive predictive value, enabling improved outcomes through earlier detection and reduced false alarms.

## Introduction

Supraventricular tachycardia (SVT) represents the predominant pathological tachyarrhythmia in the neonatal period, with an estimated incidence of 1 in 250 to 1 in 1,000 live births (1, 2). This heterogeneous group of arrhythmias accounts for approximately 90%–95% of all neonatal tachyarrhythmias and presents significant diagnostic challenges due to nonspecific symptomatic presentations and unique electrophysiological characteristics in this age group (3, 4).

The pathophysiological substrate of neonatal SVT differs substantially from older populations due to developmental immaturity of the cardiac conduction system. Atrioventricular reentrant tachycardia (AVRT) predominates, accounting for 70%–80% of cases, with accessory pathways identified in 50%–60% of neonates with SVT (5, 6). These accessory pathways demonstrate higher rates of spontaneous resolution during the first year of life compared to older children, with reported regression rates of 40%–70% (7, 8).

Clinical presentation exhibits significant heterogeneity, ranging from asymptomatic detection to profound hemodynamic compromise. Unlike older children who typically report palpitations or chest discomfort, neonates manifest nonspecific signs including tachypnea, poor feeding, irritability, and pallor (1). The relationship between symptomatology and arrhythmia duration is well-established, with approximately 50% of neonates experiencing SVT persisting >24 h developing signs of congestive heart failure, including hepatomegaly and decreased peripheral perfusion (9, 10).

Conventional diagnostic approaches rely predominantly on surface electrocardiography (ECG), which remains the gold standard for arrhythmia characterization. However, obtaining high-quality recordings in neonates presents substantial technical challenges including limited body surface area for electrode placement, frequent motion artifacts, and age-specific ECG parameters requiring specialized interpretation (11). The traditional dichotomization of tachycardia as “narrow complex” vs. “wide complex” retains utility but requires modification in the neonatal context due to naturally shorter QRS durations (12).

Recent technological advances offer unprecedented opportunities for improving SVT detection and characterization. Artificial intelligence algorithms demonstrate superior accuracy in arrhythmia detection compared to conventional automated systems (13). Advances in materials science have enabled development of biocompatible wearable monitoring solutions addressing the limitations of traditional electrode-based systems (14). Telemedicine platforms facilitate expert consultation irrespective of geographic constraints, potentially reducing diagnostic delays (15).

This review aims to systematically analyze current diagnostic approaches to neonatal SVT, critically evaluate emerging technologies, and identify specific research priorities. Understanding the comparative advantages and limitations of available diagnostic modalities is essential for optimizing management strategies and improving outcomes in this vulnerable population. While this review frequently references the neonatal intensive care unit (NICU), it is important to note that in many centers, particularly in the United States, these patients are managed in specialized pediatric cardiac intensive care unit (CICU), where multidisciplinary expertise is centralized.

## Search strategy and selection criteria

To identify relevant literature, we conducted a search of PubMed, Embase, and IEEE Xplore databases. We primarily prioritized articles published between January 2000 and September 2025 to capture contemporary diagnostic approaches and emerging technologies. Search terms included combinations of “neonatal”, “supraventricular tachycardia”, “arrhythmia”, “artificial intelligence”, “wearable sensors”, “photoplethysmography”, and “non-contact monitoring”. We prioritized studies specifically enrolling neonates (0–28 days). Foundational studies and seminal references regarding pathophysiology or incidence were included regardless of publication date. Where neonatal-specific data were limited—particularly regarding novel AI architectures and radar-based sensing—we included relevant pediatric and proof-of-concept adult studies, explicitly noting the population difference to avoid generalization bias.

## Pathophysiology and classification

The electrophysiological mechanisms underlying neonatal SVT are fundamentally influenced by developmental factors unique to this age group. The neonatal myocardium exhibits distinct electrophysiological properties including shorter action potential duration, decreased refractory periods, enhanced automaticity of subsidiary pacemakers, and the presence of transitional conduction pathways that collectively establish a substrate conducive to arrhythmogenesis (16).

## Atrioventricular reentrant tachycardia (AVRT)

AVRT constitutes the predominant mechanism in neonates, requiring an accessory pathway that provides an anatomical substrate for a macro-reentrant circuit encompassing the atria, atrioventricular node, ventricles, and the accessory pathway itself. Anatomical distribution studies demonstrate preferential localization in the left free wall (50%–60%), followed by posteroseptal (20%–30%), right free wall (10%–20%), and anteroseptal regions (5%–10%) (17). Electrophysiological characterization reveals that neonatal accessory pathways frequently exhibit decremental conduction properties that may contribute to the observed spontaneous resolution in 40%–70% of cases during the first year of life (18).

Orthodromic AVRT, characterized by antegrade conduction through the atrioventricular node and retrograde conduction through the accessory pathway, accounts for approximately 90% of AVRT cases in neonates (19). This mechanism typically produces a narrow QRS complex tachycardia with heart rates ranging from 220 to 320 beats per minute. In contrast, antidromic AVRT (antegrade conduction through the accessory pathway, retrograde through the AV node) occurs in <10% of cases and produces a wide QRS complex tachycardia that may be misdiagnosed as ventricular tachycardia (20).

## Atrioventricular nodal reentrant tachycardia (AVNRT)

AVNRT represents only 10%–15% of neonatal SVT cases, reflecting the immature development of dual AV nodal pathways in this age group (21, 22). The relative paucity of AVNRT in neonates correlates with electrophysiological studies demonstrating progressive maturation of AV nodal physiology throughout infancy and early childhood (5). When present, AVNRT in neonates typically manifests as the slow-fast variant, with antegrade conduction through the slow pathway and retrograde conduction through the fast pathway (23).

## Atrial tachycardias

Atrial tachycardias constitute approximately 10%–15% of neonatal SVT cases and encompass a heterogeneous group of arrhythmias originating from atrial tissue (24, 25). These include focal atrial tachycardia (FAT), multifocal atrial tachycardia (MAT), and atrial flutter. Focal atrial tachycardia in neonates most commonly originates from the left and right atrial appendages, pulmonary vein ostia, or the free wall (26). The underlying cellular mechanism may involve enhanced automaticity, triggered activity (early or delayed afterdepolarizations), or micro-reentry within a circumscribed area of atrial tissue (27).

## Atrial flutter

Atrial flutter in neonates represents a distinct entity characterized by a macro-reentrant circuit, typically involving the cavotricuspid isthmus. Unlike the “irregularly irregular” rhythm of atrial fibrillation, neonatal atrial flutter typically presents with a regular atrial rate of 300–500 beats per minute (bpm). Ventricular response is determined by the atrioventricular (AV) node conduction, most commonly manifesting as 2:1 conduction, though variable block can occur (28). On surface ECG, this presents as “sawtooth” flutter waves (F-waves) in inferior leads (II, III, aVF). While electrophysiologically similar to adult flutter, neonatal cases differ significantly in prognosis, often resolving without recurrence after cardioversion or short-term pharmacological control (29). These abnormalities may occur due to exposure to hypoxia, inflammatory processes, scarring, or increased pressure in cases of enlarged atrial dimensions (30).

## Clinical classification and associated conditions

The classification framework extends beyond mechanistic considerations to encompass clinical patterns and associated conditions. Incessant SVT, defined as tachycardia present for >90% of the monitored period, carries particular prognostic significance in neonates due to the elevated risk of tachycardia-induced cardiomyopathy (31). Permanent junctional reciprocating tachycardia (PJRT), a form of orthodromic AVRT involving a slowly conducting posteroseptal accessory pathway with decremental properties, represents the most common cause of incessant SVT in neonates and necessitates aggressive management to prevent cardiac dysfunction (32).

The relationship between SVT and congenital heart disease merits specific consideration in the neonatal population. Certain structural abnormalities create anatomical substrates that predispose to specific arrhythmias. Ebstein's anomaly is associated with accessory pathways in up to 10%–25% of cases, while congenitally corrected transposition of the great arteries frequently involves twin atrioventricular nodes that may serve as substrates for reentrant tachycardias (33, 34).

## Current diagnostic approaches

### Electrocardiography

**Figure 1:**
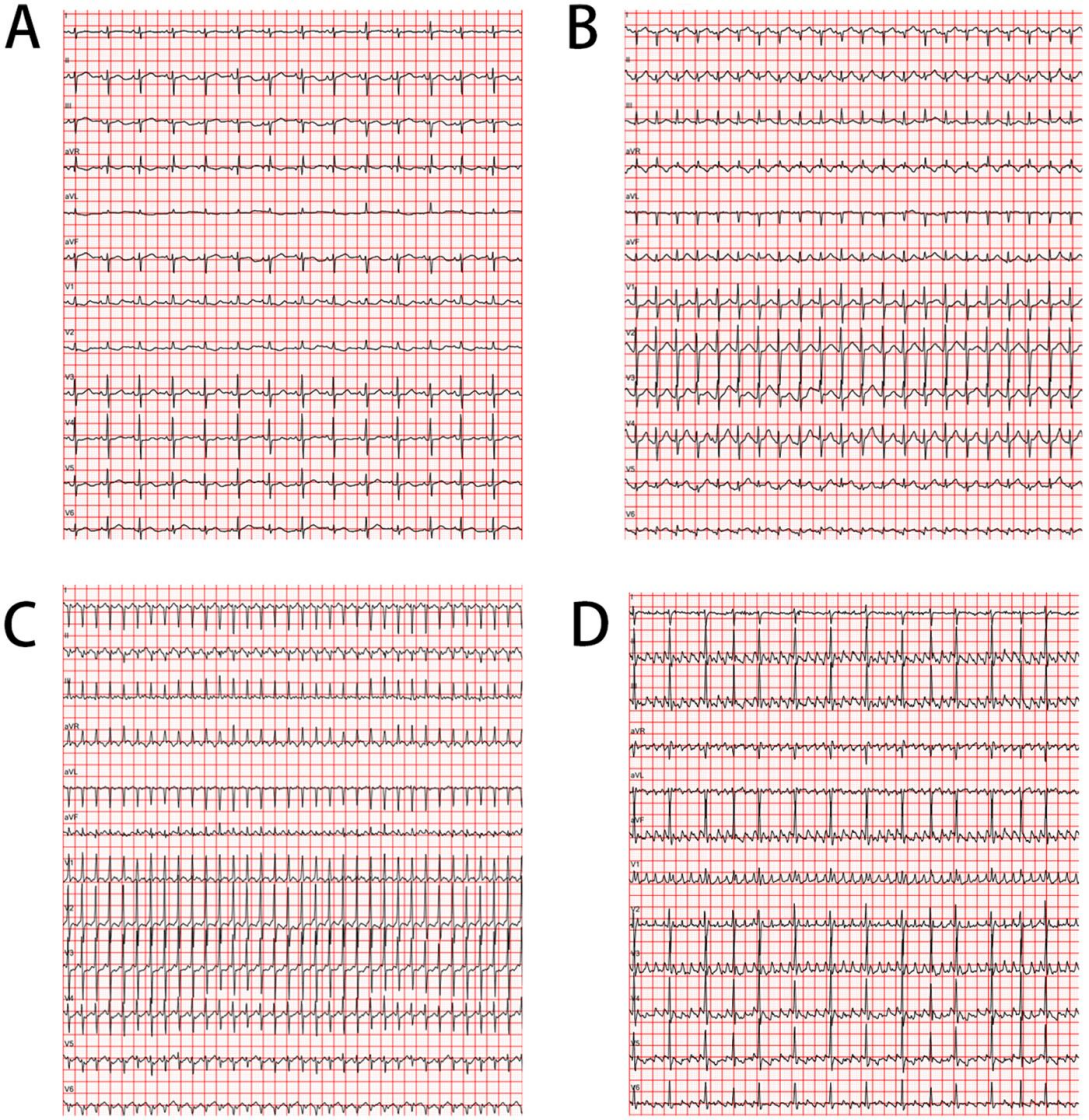
Comparative Neonatal Electrocardiogram (ECG) Tracings. **(A)** Normal Sinus Rhythm: This panel shows a normal rhythm for a neonate with a heart rate of approximately 110 beats per minute (bpm). Note the clear P wave preceding each QRS complex and the stable baseline. **(B)** Sinus Tachycardia: This panel displays a physiological rapid heart rate of around 170 bpm. While fast, it maintains a 1:1 relationship between P waves and QRS complexes, which distinguishes it from pathological tachycardias. **(C)** Supraventricular Tachycardia (Orthodromic AVRT): This panel illustrates the most common form of neonatal SVT. Key features include a very rapid heart rate (∼230 bpm), a narrow QRS complex, and the absence of visible P waves, which are often buried in the preceding T wave or ST segment. This fixed, rapid rate is a hallmark of SVT. **(D)** Atrial Flutter: This panel illustrates atrial flutter. Key features include a rapid, regular atrial rate (approx. 375 bpm) manifest as “sawtooth” flutter waves (F-waves), best visualized in inferior leads. The ventricular rhythm may be regular or slightly irregular depending on the conduction ratio (e.g., varying between 2:1 and 3:1 block), distinct from the chaotic irregularity of atrial fibrillation.

The 12-lead electrocardiogram (ECG) remains the definitive diagnostic modality for evaluating neonatal SVT, providing critical information regarding rate, rhythm, and conduction patterns (Figure 1). Diagnostic interpretation in neonates requires application of age-specific criteria, with heart rates exceeding 220–230 beats per minute strongly suggesting pathological SVT rather than physiological sinus tachycardia (35, 36). Comprehensive ECG analysis should include systematic assessment of P wave morphology, PR interval, QRS duration, and the temporal relationship between atrial and ventricular activation sequences.

In orthodromic AVRT, retrograde P waves are typically visible in the ST segment or early T wave, with an RP interval <70 milliseconds (37). The absence of pre-excitation during sinus rhythm does not exclude the diagnosis of accessory pathway-mediated tachycardia, as many accessory pathways in neonates (40%–50%) exhibit unidirectional retrograde-only conduction (38). Differential diagnosis between orthodromic AVRT and AVNRT remains challenging in neonates, as the typical discriminating features (RP interval, presence of pseudo-r’ in V1, pseudo-S in inferior leads) may be subtle or absent due to rapid heart rates (39).

Technical considerations for obtaining diagnostic-quality ECGs in neonates include modification of standard electrode placement to accommodate limited body surface area. Limb leads may be positioned proximally on the shoulders and hips rather than distal extremities to minimize motion artifact (40). Digital signal processing should minimize high-frequency filtering (0.05–250 Hz bandpass recommended) to preserve P wave morphology essential for mechanism determination (41). Paper speed adjustment to 50 mm/s, rather than the standard 25 mm/s, facilitates accurate interval measurement in rapid tachycardias (42).

### Continuous cardiac monitoring

Continuous cardiac monitoring represents a cornerstone of NICU surveillance, providing opportunities for detection of intermittent or paroxysmal arrhythmias. Contemporary monitoring systems incorporate automated arrhythmia detection algorithms based on programmable rate and regularity criteria (43). However, these systems demonstrate significant limitations in the neonatal population, with reported positive predictive values of less than 1% for SVT detection in NICU settings, highlighting the necessity for expert confirmation of automated alerts (44).

### Ambulatory monitoring

Ambulatory electrocardiographic (Holter) monitoring extends diagnostic capabilities by providing continuous recording over 24–48 h, facilitating detection of paroxysmal arrhythmias and assessment of overall arrhythmia burden (45). Technical implementation in neonates requires miniaturized recording devices and specialized electrode systems suitable for delicate neonatal skin. Digital Holter systems offer significant advantages including extended recording duration (up to 14 days), automated arrhythmia detection with adjustable sensitivity thresholds, and correlation capability between parental observations and rhythm abnormalities (46, 47).

Event recorders and implantable loop recorders provide targeted monitoring for infrequent symptomatic episodes. External loop recorders continuously record and overwrite ECG data, preserving information when activated during symptomatic periods (48). Recent development of adhesive patch-based event recorders has improved feasibility in the neonatal population by eliminating lead wires and reducing skin irritation. However, the requirement for caregiver activation during symptomatic periods represents a significant limitation, as neonates cannot communicate symptoms, and caregivers may not recognize subtle manifestations of arrhythmias (49).

### Transesophageal electrophysiology study (TEEPS)

TEEPS offers a minimally invasive methodology for detailed characterization of SVT mechanisms in neonates. This technique involves positioning an electrode catheter in the esophagus adjacent to the left atrium, providing high-resolution recording of atrial electrical activity (50). TEEPS can determine the tachycardia mechanism, assess accessory pathway properties, and evaluate antiarrhythmic drug efficacy. The procedure can be performed at the bedside without sedation in most neonates, though careful attention to catheter size selection (4–5 Fr) and insertion depth calculation based on body length is essential (51, 52).

### Echocardiographic assessment

Echocardiographic assessment serves multiple functions in neonatal SVT evaluation. Echocardiographic assessment serves multiple functions in neonatal SVT evaluation. Beyond exclusion of structural cardiac abnormalities, contemporary imaging techniques including tissue Doppler imaging and strain analysis can detect subtle abnormalities in ventricular function resulting from chronic tachycardia (53). Quantitative parameters including left ventricular ejection fraction, myocardial performance index (Tei index), and global longitudinal strain provide sensitive indicators of tachycardia-induced myocardial dysfunction (54). In cases of suspected PJRT, advanced echocardiographic techniques including tissue Doppler may occasionally identify posteroseptal accessory pathway insertion sites (55).

### Laboratory investigations

Laboratory investigations play a supportive role in comprehensive SVT evaluation. Electrolyte abnormalities, particularly of potassium, calcium, and magnesium, can precipitate or exacerbate arrhythmias and warrant systematic assessment (56). Thyroid function testing is indicated given the established association between neonatal hyperthyroidism and SVT, particularly in infants of mothers with autoimmune thyroid disease (57). Cardiac biomarkers including troponin I/T and N-terminal pro-B-type natriuretic peptide (NT-proBNP) may be elevated in cases of tachycardia-induced cardiomyopathy and provide objective assessment of disease severity and treatment response (58).

### Integrated diagnostic approach

The integration of multiple diagnostic modalities is frequently necessary for comprehensive evaluation of neonatal SVT. A staged diagnostic approach, beginning with clinical assessment and 12-lead ECG, followed by continuous monitoring and specialized techniques as clinically indicated, maximizes diagnostic yield while minimizing invasive procedures. The selection of specific diagnostic modalities should be individualized based on clinical presentation, suspected arrhythmia mechanism, frequency of episodes, and hemodynamic stability.

### Comparative utility of diagnostic modalities

The selection of diagnostic tools in neonates requires balancing diagnostic yield with invasiveness. 12-lead ECG remains the gold standard for confirmatory diagnosis but is limited by its snapshot nature, often missing paroxysmal events. Continuous bedside telemetry serves primarily as a screening tool for safety but suffers from low specificity and high false-alarm rates. Ambulatory (Holter) monitoring is critical for quantifying arrhythmia burden and correlating symptoms with rhythm, though it is constrained by skin fragility and lead displacement in active neonates. TEEPS provide definitive substrate characterization and mechanism differentiation (e.g., distinguishing AVRT from atrial flutter) with high sensitivity, but are invasive and require specialized expertise.

## Emerging technologies

### Artificial intelligence and machine learning

Artificial intelligence (AI) and machine learning (ML) algorithms represent a paradigm shift in automated arrhythmia detection and classification. Unlike conventional rule-based algorithms that rely on predetermined thresholds and pattern recognition, ML systems can identify complex, nonlinear relationships in high-dimensional data and adapt to individual patient characteristics (59). Deep neural networks, particularly convolutional neural networks (CNNs) and recurrent neural networks (RNNs), have demonstrated exceptional accuracy in ECG interpretation, with multiple studies reporting performance equivalent to or exceeding that of board-certified cardiologists (13, 60).

In the context of pediatric arrhythmias, including neonatal cases, age-specific AI models trained on dedicated pediatric ECG databases have achieved sensitivity and specificity exceeding 90% for certain arrhythmia detection (61). These algorithms demonstrate advantages in reducing false-positive alerts in monitoring systems, with the potential to differentiate pathological rhythms from physiological ones (62). Advanced deep learning models can identify pre-excitation patterns that may be missed by human interpreters, particularly in pediatric cases with subtle delta waves or accessory pathways (63). It is important to note, however, that these performance metrics are largely derived from mixed pediatric populations. Dedicated validation specifically within the first 28 days of life remains limited, where rapid heart rates and distinct signal-to-noise ratios may affect algorithm performance.

Implementation of AI algorithms in clinical environments requires attention to computational requirements and integration with existing monitoring infrastructure. Edge computing solutions, which process data locally rather than relying on cloud-based systems, enable real-time analysis while maintaining data security and minimizing latency (64). Contemporary commercial monitoring platforms increasingly incorporate ML-based arrhythmia detection, though specific validation in neonatal populations remains limited (61).

The development of explainable AI models addresses concerns regarding the “black box” nature of deep learning systems. These models provide visual representations of ECG features contributing to diagnostic decisions, enhancing clinician trust and facilitating educational opportunities (65). For neonatal SVT, explainable AI can highlight specific P wave morphologies, PR relationships, or subtle rate variations supporting the diagnosis, improving clinician understanding and diagnostic accuracy (66).

### Wearable and wireless monitoring technologies

Wearable and wireless monitoring technologies address fundamental limitations of conventional electrode-based systems. Advanced materials science has enabled development of flexible bioelectronics that conform to neonatal anatomy while maintaining diagnostic signal quality (67). Smart textile technologies integrate conductive fibers directly into garments, creating comfortable, washable monitoring solutions that maintain consistent electrode contact without adhesives (68). Systematic evaluation demonstrates that these systems provide diagnostic-quality ECG recording comparable to conventional systems while significantly reducing skin complications and improving infant mobility.

Wireless patch monitors represent another significant advance in neonatal cardiac monitoring. These single-use devices typically incorporate ECG electrodes, signal processing electronics, and wireless transmission capabilities in a waterproof patch smaller than a standard adhesive bandage (49). Contemporary devices provide up to 30 days of continuous monitoring with automated arrhythmia detection and wireless transmission capabilities. Clinical studies in pediatric populations demonstrate high diagnostic yield for detecting intermittent arrhythmias, with patient compliance exceeding 95% due to improved comfort and convenience compared to traditional Holter monitors (69).

The integration of photoplethysmography (PPG) technology in wearable devices offers complementary rhythm detection capabilities. While PPG cannot provide the detailed electrical information of ECG, advanced signal processing algorithms can accurately detect heart rate and rhythm irregularities from optical measurements of pulsatile blood volume changes (70). Multi-modal monitoring combining ECG and PPG improves detection accuracy by providing physiological redundancy in cases of electrode displacement or signal artifact (71).

### Advanced signal processing

Advanced signal processing techniques enhance the quality and interpretability of neonatal ECG recordings. Adaptive filtering algorithms effectively remove motion artifact and baseline wander while preserving diagnostic ECG features, addressing a major challenge in neonatal monitoring (72). These algorithms utilize accelerometer data from wearable devices to identify periods of movement and apply appropriate filtering strategies. Wavelet transformation techniques provide superior time-frequency analysis of ECG signals, facilitating detection of transient rhythm abnormalities (73).

Compressed sensing methodologies enable efficient data transmission and storage while maintaining diagnostic quality, particularly important for continuous long-term monitoring (74). By exploiting the sparse nature of arrhythmia events in the temporal domain, these methods achieve compression ratios exceeding 10:1 without clinically significant information loss. Signal quality indices (SQI) provide real-time assessment of ECG signal quality, allowing automated systems to identify periods of suboptimal recording and prevent false arrhythmia alerts (75).

### Telemedicine integration

Telemedicine integration with cardiac monitoring systems enables expert consultation and real-time decision support for neonatal SVT management. Secure cloud-based platforms facilitate transmission of ECG data to pediatric electrophysiology specialists, enabling timely diagnosis and treatment recommendations regardless of geographic constraints (76). Mobile health applications designed for caregivers provide interfaces for symptom documentation, medication adherence monitoring, and direct communication with healthcare providers (77).

### Novel sensing modalities

Novel sensing modalities extend beyond traditional electrical measurements to offer new approaches to cardiac rhythm assessment. Ballistocardiography (BCG) and seismocardiography (SCG) detect mechanical vibrations associated with cardiac contraction, providing contactless monitoring options for neonates with fragile skin conditions or those requiring minimal handling (78, 79). Radar-based vital sign monitoring employs low-power microwave signals to detect chest wall movement associated with cardiac and respiratory activity. Recent technological iterations demonstrate accurate heart rate detection in neonates using systems integrated into incubators, eliminating the requirement for direct skin contact (80).

Magnetocardiography (MCG), which detects the weak magnetic fields generated by cardiac electrical activity, offers superior signal-to-noise ratio compared to surface ECG, particularly for fetal and neonatal applications (81, 82). While conventional MCG systems require specialized magnetically shielded environments, advancements in quantum sensing technology utilizing optically pumped magnetometers (OPMs) promise portable magnetocardiography systems suitable for bedside clinical application (83).

### Clinical applications and real-world implementation

The translation of diagnostic approaches and emerging technologies into clinical practice for neonatal SVT requires careful consideration of patient-specific factors, resource availability, and multidisciplinary collaboration. In real-world settings, initial evaluation typically begins in NICU or a pediatric CICU, or in the emergency department, where prompt recognition of SVT is critical to prevent hemodynamic deterioration.

### Acute management protocols

Clinical management relies on rapid risk stratification. For hemodynamically stable infants, vagal maneuvers or adenosine remain first-line interventions (84). Chronic management strategies are increasingly influenced by diagnostic precision; the use of continuous monitoring and improved arrhythmia burden assessment can guide the titration of antiarrhythmic agents (e.g., beta-blockers like propranolol or class Ic agents), potentially allowing for earlier weaning in patients with low arrhythmia burden (85). The choice of agent should be tailored to the specific SVT mechanism identified via ECG or TEEPS.

### Technology integration in clinical practice

Integration of emerging technologies enhances diagnostic precision and monitoring efficiency in clinical environments. AI-driven ECG analysis has been implemented in NICU settings to reduce false alarms from conventional monitors, with convolutional neural networks achieving over 90% accuracy in differentiating SVT from sinus tachycardia by analyzing subtle waveform patterns (86). Wearable wireless patches and non-contact radar-based systems enable continuous, unobtrusive heart rate variability monitoring, facilitating early detection of arrhythmia episodes in high-risk neonates without skin irritation or motion artifacts (87).

A pediatric telecardiology system that integrates community-based primary care with hospital services has proven valuable for transmitting and reporting ECGs. This approach helps reduce unnecessary hospital referrals, especially for routine pre-participation sports screenings, and has demonstrated high user satisfaction due to time savings and avoiding hospital visits (88). In a 10-year institutional review, such integrated approaches contributed to zero mortality and low rehospitalization rates (21%) among 38 neonates with SVT, highlighting improved outcomes through protocolized care (89).

### Implementation challenges

Despite these advances, real-world implementation faces significant challenges. Diagnostic delays often occur due to nonspecific presentations mimicking sepsis or feeding issues, necessitating heightened clinical vigilance and multidisciplinary input from neonatologists, cardiologists, and nurses (90). For AI technologies, barriers include data biases from underrepresented populations, lack of neonatal-specific validation datasets, and “black box” interpretability issues that erode clinician trust (86). Ethical considerations, such as ensuring equity in algorithm training to avoid exacerbating racial disparities in outcomes, further complicate adoption.

Wearable and non-contact devices, while promising, require robust calibration to mitigate interference from ambient factors like lighting or movement, with current systems showing variable accuracy in preterm infants (87). Financial constraints, including the absence of reimbursement codes for AI-assisted monitoring, limit widespread deployment, particularly in low-resource settings.

### Clinical outcomes and future strategies

Outcomes from real-world applications demonstrate substantial benefits when these challenges are addressed. Prophylactic antiarrhythmic regimens have shortened hospital stays to an average of 15 days, with most neonates achieving spontaneous resolution by 12 months (85). Emerging tools like heart rate characteristics (HRC) scoring via AI have reduced sepsis-related mortality by 20%–40% in NICUs by enabling proactive interventions, suggesting similar potential for SVT management (86).

Future implementation strategies should prioritize multicenter collaborations for data standardization, prospective trials validating AI in diverse cohorts, and training programs to enhance clinician proficiency. By bridging these gaps, diagnostic modalities can be optimized to minimize morbidity and improve long-term cardiac health in neonates with SVT.

## Challenges and future directions

### Technical limitations

Despite significant advances in diagnostic technologies for neonatal SVT, numerous challenges and limitations persist that must be addressed to optimize clinical implementation and patient outcomes. Technical limitations of current technologies include signal artifacts from patient movement, skin fragility affecting electrode adhesion duration, and power management constraints in wearable devices (87). The high baseline heart rates, rapid respiratory rates, and frequent movement characteristic of neonates create complex signal processing challenges that even sophisticated algorithms struggle to resolve completely (86).

### Algorithm development challenges

Algorithm development faces fundamental obstacles including limited availability of annotated training data, with most institutions possessing only small datasets of confirmed neonatal SVT cases. This data scarcity is compounded by the heterogeneity of SVT mechanisms and presentations in neonates. Annotation quality represents another critical challenge, with inter-observer variability in ECG interpretation among pediatric electrophysiologists noted in related arrhythmia studies (91).

The relative opacity of deep learning algorithms raises concerns regarding clinical trust, interpretability, and medicolegal liability. While explainable AI techniques continue to evolve, they frequently provide *post-hoc* rationalizations rather than transparent insight into algorithmic decision-making processes. This limitation becomes particularly problematic when algorithms generate unexpected or counterintuitive diagnoses that clinicians must act upon in time-sensitive clinical scenarios.

### Generalization and validation issues

Algorithm generalization across diverse populations remains suboptimal. Systems trained predominantly on data from tertiary care centers in high-income countries may perform poorly in different clinical settings or demographic populations. Variations in ECG equipment specifications, recording protocols, and patient demographics significantly impact algorithm performance, necessitating extensive external validation before widespread clinical deployment (92).

### Clinical integration barriers

Clinical integration faces significant resistance due to workflow disruption and alert fatigue from existing monitoring systems. Studies demonstrate that nurses in intensive care settings like the NICU and CICU experience numerous monitoring alarms per shift, with a low proportion representing clinically actionable events. Implementation of new technologies must demonstrate substantial improvements in specificity to overcome existing skepticism toward automated alert systems (93).

Interoperability between monitoring systems and electronic health records creates information silos that impede comprehensive patient evaluation. Data from proprietary wearable devices frequently cannot be integrated directly into institutional electronic health records, necessitating manual transcription or parallel documentation systems (94). This fragmentation increases error potential and limits the capabilities for automated clinical decision support.

### Regulatory and economic challenges

Regulatory uncertainty regarding AI-based medical devices delays clinical implementation. The FDA's evolving approach to Software as a Medical Device (SaMD) requires continuous algorithm validation, creating tension with traditional approval processes designed for static medical devices (95). This regulatory ambiguity delays market entry for innovative technologies and increases development costs.

The digital divide and socioeconomic disparities threaten equitable access to advanced monitoring technologies. Families without reliable internet connectivity or smartphone access cannot fully utilize home monitoring systems with remote transmission capabilities. This disparity disproportionately affects rural and lower socioeconomic populations already encountering barriers to specialized pediatric cardiac care.

### Future research priorities

Future research priorities must address these challenges through coordinated multidisciplinary efforts. Development of federated learning approaches enables algorithm training across multiple institutions while preserving data privacy and security. This methodology could address the current limitation of small training datasets by combining anonymized data from dozens of NICUs worldwide without transferring protected health information across institutional boundaries (96).

Next-generation sensing technologies that eliminate direct skin contact offer promising solutions for current limitations in electrode-based monitoring. Ultra-wideband radar systems capable of detecting cardiac motion through clothing or incubator walls could enable continuous monitoring without skin irritation or electrode displacement (87). Similarly, optical sensing technologies utilizing hyperspectral imaging can simultaneously assess tissue oxygenation, perfusion, and cardiac rhythm from a single non-contact sensor, further highlighting the potential for electrode-free neonatal monitoring.

### Personalized medicine and digital twins

Looking toward future horizons, “Digital Twin” technology—computational models simulating individual patient physiology—offers a theoretical framework for precision management. While currently in proof-of-concept stages for pediatric electrophysiology, future iterations could potentially simulate a neonate's specific conduction properties to predict responses to antiarrhythmic drugs before administration (97). However, this technology remains experimental. Implementation in neonatology would require overcoming significant challenges in modeling the rapidly maturing neonatal myocardium and validating these simulations against clinical outcomes.

### Implementation science and infrastructure development

Implementation science research specific to neonatal intensive care environments is essential for successful technology adoption. Systematic investigation of workflow integration, staff training methodologies, and organizational factors influencing implementation success could guide deployment strategies. Understanding institutional characteristics that facilitate or impede technology adoption could inform best practices for widespread implementation.

The development of comprehensive neonatal ECG databases with expert annotation and standardized quality metrics represents a critical research infrastructure need. These resources must include diverse populations, multiple recording systems, and standardized outcome measures to enable robust algorithm validation. International collaboration on data standardization and sharing protocols would accelerate progress while ensuring global applicability of research findings.

### Ethical considerations

The integration of AI into neonatal care necessitates a robust ethical framework. A primary concern in the NICU setting is the “black box” nature of deep learning; clinicians may face dilemmas when an algorithm predicts an arrhythmia or deterioration without a visible ECG correlate. Furthermore, algorithms trained predominantly on data from large, tertiary academic centers may not generalize to diverse populations, potentially exacerbating healthcare disparities. Ethical implementation requires ensuring that training datasets are representative of the diverse genetic and physiological profiles seen in global neonatal populations.

## Conclusion

Neonatal SVT diagnosis stands at a transformative juncture where traditional clinical approaches intersect with revolutionary technological innovations. This comprehensive review has examined the current diagnostic landscape, emerging technologies, and future directions that collectively promise to enhance outcomes for this vulnerable population.

The integration of artificial intelligence, wearable monitoring technologies, and advanced signal processing techniques has demonstrated tangible clinical benefits, including reduced time to diagnosis, decreased length of hospitalization, and improved detection of intermittent arrhythmias. Early implementation experiences provide evidence supporting the potential of these technologies to transform neonatal arrhythmia management.

However, significant challenges persist, including technical limitations, implementation barriers, regulatory uncertainty, and concerns regarding healthcare disparities. Addressing these challenges requires coordinated multidisciplinary efforts spanning algorithm development, validation methodology, clinical workflow integration, and ethical framework establishment.

The future of neonatal SVT management lies not in replacing clinical expertise with technology, but in creating synergistic partnerships between advanced computational approaches and experienced clinicians. By focusing on patient-centered outcomes while embracing responsible innovation, the medical community can significantly improve care delivery and outcomes for neonates with supraventricular tachycardia.

Success will ultimately be measured not by technological sophistication, but by tangible improvements in diagnostic accuracy, treatment optimization, patient outcomes, and healthcare equity. The convergence of multiple technological advances creates unprecedented opportunities to address longstanding challenges in neonatal arrhythmia diagnosis, but realizing this potential requires sustained commitment to rigorous validation, equitable implementation, and ethical application.
